# DNA Vaccines Targeting Novel Cancer-Associated Antigens Frequently Expressed in Head and Neck Cancer Enhance the Efficacy of Checkpoint Inhibitor

**DOI:** 10.3389/fimmu.2021.763086

**Published:** 2021-10-18

**Authors:** Chuan Wang, Nur Syafinaz Zainal, San Jiun Chai, James Dickie, Chai Phei Gan, Natasha Zulaziz, Bryan Kit Weng Lye, Ruhcha V. Sutavani, Christian H. Ottensmeier, Emma V. King, Mannil Thomas Abraham, Siti Mazlipah binti Ismail, Shin Hin Lau, Thomas George Kallarakkal, Kein Seong Mun, Rosnah binti Zain, Zainal Ariff Abdul Rahman, Gareth J. Thomas, Sok Ching Cheong, Natalia Savelyeva, Kue Peng Lim

**Affiliations:** ^1^ Head and Neck Cancer Center, Institute of Systems, Molecular and Integrative Biology, University of Liverpool, Liverpool, United Kingdom; ^2^ Cancer Immunology and Immunotherapy Unit, Cancer Research Malaysia, Selangor, Malaysia; ^3^ Cancer Sciences, University of Southampton, Southampton, United Kingdom; ^4^ Ministry of Health Malaysia, Department of Oral & Maxillofacial Surgery, Tengku Ampuan Rahimah Hospital, Klang, Malaysia; ^5^ Department of Oral and Maxillofacial Clinical Sciences, Faculty of Dentistry, University of Malaya, Kuala Lumpur, Malaysia; ^6^ Stomatology Unit, Cancer Research Centre, Institute for Medical Research, Kuala Lumpur, Malaysia; ^7^ Oral Cancer Research and Coordinating Centre, Faculty of Dentistry, University of Malaya, Kuala Lumpur, Malaysia; ^8^ Department of Pathology, Faculty of Medicine, University of Malaya, Selangor, Malaysia; ^9^ Faculty of Dentistry, Malaysian Allied Health Sciences Academy (MAHSA) University, Selangor, Malaysia; ^10^ Department of Oral and Maxillofacial Clinical Sciences, Faculty of Dentistry, University Teknologi Majlis Amanah Rakyat (MARA), Selangor, Malaysia

**Keywords:** DNA vaccine, cancer immunotherapy, head and neck cancer, MAGED4B, FJX1, cancer antigens

## Abstract

HPV-independent head and neck squamous cell carcinoma (HNSCC) is a common cancer globally. The overall response rate to anti-PD1 checkpoint inhibitors (CPIs) in HNSCC is ~16%. One major factor influencing the effectiveness of CPI is the level of tumor infiltrating T cells (TILs). Converting TILlow tumors to TILhigh tumors is thus critical to improve clinical outcome. Here we describe a novel DNA vaccines to facilitate the T-cell infiltration and control tumor growth. We evaluated the expression of target antigens and their respective immunogenicity in HNSCC patients. The efficacy of DNA vaccines targeting two novel antigens were evaluated with or without CPI using a syngeneic model. Most HNSCC patients (43/44) co-expressed MAGED4B and FJX1 and their respective tetramer-specific T cells were in the range of 0.06-0.12%. In a preclinical model, antigen-specific T cells were induced by DNA vaccines and increased T cell infiltration into the tumor, but not MDSC or regulatory T cells. The vaccines inhibited tumor growth and improved the outcome alone and upon combination with anti-PD1 and resulted in tumor clearance in approximately 75% of mice. Pre-existence of MAGED4B and FJX1-reactive T cells in HNSCC patients suggests that these widely expressed antigens are highly immunogenic and could be further expanded by vaccination. The DNA vaccines targeting these antigens induced robust T cell responses and with the anti-PD1 antibody conferring excellent tumor control. This opens up an opportunity for combination immunotherapy that might benefit a wider population of HNSCC patients in an antigen-specific manner.

## Introduction

HNSCC is the sixth most common cancer in the world and is more prevalent in South East Asia where it is the second most common malignancy (1). The incident in some cases is linked to HPV but for HPV independent (HPV^neg^) subgroup which represents 60-80% of all HNSCC cases, the causes are unclear and often linked to consumption of alcohol, smoking, smokeless tobacco and bethel quid [reviewed in (2)]. The HPV^neg^ HNSCC is often difficult to treat; surgery and radiotherapy are associated with significant morbidity and a relatively static 5-year survival rate of around 50-60% (3).

Immune checkpoint inhibitors prolonged survival of some patients with HNSCC and have now gained the approval for subsets of patients with advanced disease, however the responses have only been observed in 14-25% of patients (4–6). While the response to checkpoint inhibitors is associated with many factors including expression of PD-L1 (7, 8), tumor mutational burden (9) and the interferon gene signature (10), one major factor is the presence of pre-existing tumor-infiltrating T cells (TIL) (11–15). High TIL levels correlate with significantly longer survival, and many patients who respond to checkpoint inhibitors fall into this category. TILlow tumors, including most HPV^neg^ HNSCC, are commonly categorized as ‘immune desert’ (absent T cells) or ‘immune excluded’ (excluded T cells) (13, 16, 17). This emphasizes the urgent need of novel immunotherapies that can work in these patients. One such intervention is to use cancer vaccines to induce or expand specific T cell responses against cancer.

For HPV positive (HPV^pos^) HNSCC, cancer vaccines targeting oncoprotein E6 and E7 from HPV have induced a durable anti-tumor response (18, 19). For HPV^neg^ HNSCC such viral antigens are not available. Targeting mutated antigens (neo-antigens) is an emerging strategy and has demonstrated clinical benefits in late-stage melanoma and newly diagnosed glioblastoma in combination with checkpoint inhibitors (20–23). A mutanome-targeting DNA vaccine has entered a phase 1/2 trial for patients with locally advanced or metastatic solid tumors who received checkpoint inhibitor treatment but did not reach a complete response (24). However, the mutanome-targeting approach is time consuming and costly as it requires the generation of personalized vaccines for each patient. We therefore focused on developing cancer vaccines targeting tumor associated antigens (TAAs) in order to generate off-the-shelf treatment to a large proportion of HNSCC patients. Recently the feasibility of this approach has been demonstrated in patients with unresectable melanoma post-anti-PD1 treatment. Antigen-specific polyfunctional T-cells were induced in most patients, and some patients had a partial response or stable disease (25).

We have previously shown that MAGED4B and FJX1 are over-expressed in a small cohort of primary HNSCC patients in Malaysia (26, 27). Here we sought to develop a feasible therapeutic strategy by targeting these antigens in HNSCC globally. To achieve this, we first investigated their expression pattern and immunogenicity in HNSCC patient cohorts from Malaysia and the UK. We then designed DNA vaccines targeting MAGED4B and FJX1. We demonstrated that our novel DNA vaccine monotherapy delayed tumor growth in a mouse model expressing the target antigens and increased T cell infiltration into the tumor bed. Promisingly, our vaccine acted synergistically when co-administered with anti-PD1 treatment resulting in tumor clearance in a large proportion of mice.

## Materials and Methods

### Patient Sample

Samples from two cohorts of patients were processed at two separate facilities following surgery. For the Malaysian cohort, blood samples from 28 HNSCC patients were collected from the University Malaya Medical Centre, Tengku Ampuan Rahimah Hospital and Hospital Kuala Lumpur. Since HPV positive HNSCC is rare in Malaysia (28), these patients were not tested for HPV. For the UK cohort, blood samples from 21 HPV^neg^ HNSCC patients were collected from patients treated at Poole Hospital at the time of surgery. The samples were both primary and recurrent tumors. The demographic and clinico-pathological information of 49 HNSCC patients from Malaysia and UK are presented in **Table 1**. Peripheral blood mononuclear cells (PBMC) were isolated from whole blood. HLA-A status for the Malaysian cohort was determined by direct staining of PBMC with mouse anti-human HLA-A2-PE and HLA-ABC-PE (BD Biosciences, US). HLA status for the UK cohort was determined using the AllSet Gold HLA-A SSP Kit (One Lambda, US). Formalin-fixed paraffin-embedded (FFPE) tissues from HNSCC patients that are in excess of diagnosis were identified and sectioned into 4µm thick for hematoxylin and eosin (H&E), as well as immunohistochemistry (IHC) staining. For tissues from normal organs, 3-4 independent tissue microarrays generated in-house were analyzed for the expression of MAGED4B and FJX1.

**Table 1:** The demographic and clinico-pathological information of 49 HNSCC patients included in this study.

Variables		Sample size, n	Percentage, %
Age (Median=64, Range=13-91)			
	<64	24	49.0
	≥64	25	51.0
Gender			
	Male	32	65.3
	Female	17	34.7
Ethnicity			
	Malay	2	4.1
	Chinese	9	18.4
	Indian	17	34.7
	Caucasian	21	42.9
Staging			
	Early (I & II)	16	32.7
	Late (III & IV)	32	65.3
	Not available	1	2.0
Risk habits			
	Smoking	11	22.4
	Alcohol drinking	2	4.1
	Betel quid chewing	7	14.3
	More than 1 risk habits	15	30.6
	No risk habits	9	18.4
	Not known	5	10.2
MAGED4B expression			
	Positive	44	100.0
	Negative	0	0.0
	Samples not available*	5	–
FJX1 expression			
	Positive	43	97.7
	Negative	1	2.3
	Samples not available*	5	–

*Formalin-fixed paraffin-embedded tissue samples not available hence staining was not performed on these patients.

This project was approved by the Institutional Review Board of the Faculty of Dentistry, University of Malaya [DFOS1706/0026(L)], Medical Research and Ethics Committee, Ministry of Health, Malaysia (NMRR-16-2638-33703) and UK Medical Research and Ethics Committee and by institutional approval at the Southampton University Hospitals NHS Foundation Trust, Southampton, UK (REC No. 09/H0501/90). Written informed consent was obtained from participating patients prior to sample collection.

### Peptides

MAGED4B and FJX1 overlapping peptide pools (OPP) consisting of 183 and 107 15-mers peptides with 11 amino acids overlap respectively. CEFT peptide pool consists of MHC class I and II peptides from cytomegalovirus (CMV), Epstein-Barr Virus (EBV), Influenza A and Clostridium tetani was used as positive control in the *in vitro* stimulation (IVS) ELISpot assay. All peptides were produced by JPT Peptide Technologies (Germany) with >70% purity by high-performance liquid chromatography (HPLC).

### *In Vitro* Stimulation of T Cells

PBMCs were thawed and rested at high density (1x10^7^ cells per well of a 24-well plate) for two days before T cells were isolated using CD4+ and CD8+ microbeads (Miltenyi, UK). The remaining T-cell depleted PBMCs were transfected with mRNA encoding individual antigens. DNA fragments encoding each antigen (MAGED4B, FJX1 or CEFT) were synthesised through the Invitrogen GeneArt service (ThermoFisher, UK) and inserted into the pcDNA3. The DNA sequence synthesised for each antigen consisted of the antigen sequence, flanked at the N-terminus with an Ig signalling peptide and at the C terminus with an MHC-I trafficking signal (MITD) (29). mRNA for each antigen was produced using the HiScribe T7 ARCA mRNA kit with tailing from linearized plasmids (New England Biolabs, UK). Five micrograms of mRNA encoding CEFT or 10µg of MAGED4B or FJX1 mRNA was used to transfect T-cell depleted PBMC using the Human Dendritic Cell Nucleofector kit (Lonza, UK) to serve as antigen-presenting cells (APC). Purified T cells were incubated with transfected and irradiated (15 Gy) APC (ratio1:1) in OpTmizer™ medium (Thermo, UK) supplemented with 20 U/ml IL-2 and 5 ng/ml IL-15 (both PeproTech, US) at 37°C for 14 days. Half of the medium was changed to fresh OpTmizer medium supplemented with 20 U/ml IL-2 every 2-3 days. Stimulated T cells were subjected to ELISpot.

### Human IFNγ ELISpot Assay

1 x 10^4^ stimulated T cells were incubated with peptide-loaded dendritic cells (DCs) at 1:30 ratio on a 96 well ELISpot plates pre-coated with anti-human IFNγ antibody (mAb 1-DIK, Mabtech, UK) overnight at 37°C. DC were differentiated from autologous CD14+ monocytes, according to Miltenyi manufacturer’s protocol in ImmunoCult™ DC differentiation media (Stemcell Technologies, UK) for two weeks. DCs were loaded with 1 µg/ml CEFT, MAGED4B or FJX1 OPP. Samples were plated in duplicate or triplicate. Spots were detected with a biotin-conjugated human IFNγ antibody (mAb 7-B6-1, Mabtech UK) followed by incubation with Streptavidin ALP (Mabtech, UK) and BCIP/NBT (Thermo, UK). Plates were scanned using ImmunoSpots reader (AID, UK). The mean values were represented as spot-forming cells (SFCs) per 1 x 10^4^ T cells. Levels were considered positive if at least two times above medium control.

### Flow Cytometry Analysis of Human Samples

To detect antigen-specific T cells, PBMC from Malaysian HLA-A2 patients were first incubated with FVS780 (fixable viability stain) (BD Biosciences, US) for 15 min at room temperature (RT), washed and further incubated with MAGED4B^501-509^/HLA-A2 tetramer-PE for 30 min at RT. After incubation, cells were stained with anti-CD3+ (FITC: SK7), CD4+ (PerCP Cy5.5:SK3) and CD8+ (BV510: RPA-T8) for 30 min at RT. For UK cohort, similar protocol was used to detect antigen-specific T cells. PBMCs were first stained with Live/dead violet stain (Invitrogen, US), followed by MAGED4B^501-509^/HLA-A2 tetramer-PE, anti-CD3+ (FITC: OKT3), CD4+ (APC: OKT4), CD8+ (PE-Cy7: SK1) for 30 min at RT. MAGED4B^501-509^/HLA-A2 tetramer-PE was generated in house by University of Southampton Protein Core Facility. Gating strategy is detailed in **Figure S1A**.

All stained cells were analyzed using LSR Fortessa flow cytometer or FACSCanto (both BD Biosciences, US) and gated against fluorescence minus one control (FMO) or unstained controls. The flow panels were designed to accommodate different lasers and FACS machine capacities in both institutions. Details of all antibodies used in the flow cytometry staining are provided in **Table S1**. Analysis was performed using FlowJo software (BD Biosciences, US).

### Generation of pDom Fusion DNA Vaccines

The human wild type sequences of MAGED4B and FJX1 were cloned separately into the pcDNA3.1 (Invitrogen, UK) plasmid as a fusion with the Dom sequence from fragment C of tetanus toxin as source of CD4+ T cell help (30). The sequence encoding mus IgH signal peptide MGWSCIIFFLVATATGVHS was inserted at the N-terminus of each construct to enhance secretion. DOM fragment and the antigen of interest MAGED4B or FJX1 were separated with a seven amino acid linker (AAAGPGP).

DNA sequencing was performed to confirm that the plasmids contained the correct DNA construct. The generated plasmid DNA fusions referred as pDom-MAGED4B (pDom-M) and pDom-FJX1 (pDom-F) (plasmid constructs in **Figure S2** pDom plasmid without the insertion of antigens was used as vector control. All plasmids were propagated in DH-5α and purified using QIAGEN Plasmid Plus Giga Kit (QIAGEN, US), according to manufacturer’s instruction.

### Animal Study

All procedures involving the use of animals were reviewed and approved by the Animal Ethics Committee of Universiti Kebangsaan Malaysia (Approval No. CRM/2017/KUE PENG/25-JAN./820-APR.-2017-APR.-2020) and Animal Ethics Committee of University of Southampton under the Project Licence (P8969333C). All animal experiments complied with the National Institute of Health guide for the care and use of Laboratory animals (NIH Publications No. 8023, revised 1978).

### Immunogenicity of pDom-M/F *In Vivo*

C57BL/6 and HLA-A2 tg mice (transgenic for the HLA-A2.1 allele, HHD) (31, 32) used in the immunogenicity study were bred in animal facility of the University Southampton. C57BL/6 or HLA-A2 tg mice aged 6 -10 weeks (n=5/group) received either 50μg pDom-M, pDom-F or pDom vector control vaccines intramuscularly (i.m.) into each quadriceps muscles of the hind legs. C57BL/6 mice received a booster injection with an equivalent amount of the vaccine after one week, while HLA-A2 tg mice received a booster injection after 3 weeks with electroporation (EP). EP was carried out on mice anaesthetized by isofluorane, using a custom-made pulse generator from Inovio Pharmaceuticals, as described previously (33).

At endpoint, spleens were harvested from experimental mice, and subsequently mashed though 70 µm strainer to obtain a single cell suspension. Lymphocytes were then isolated from splenocytes using Lymphoprep™ (STEMCELL, UK) following manufacturer’s protocol. To determine the presence of IFNγ secreting lymphocytes, anti-mouse IFNγ ELISpot kit (BD Bioscience, UK) was used according to manufacturer’s protocol. Mouse lymphocytes (2.5x10^5^ cells/well) were incubated in complete RPMI media with no peptide (background control), p30 control peptide (1µM), MAGED4B or FJX1 OPP (1µM each peptide) on pre-coated anti-mouse IFNγ ELISpot plates for 40h at 37°C. Samples were plated in triplicate. Spots were detected with a biotin-conjugated mouse IFNγ antibody (BD Bioscience, UK) followed by incubation with Streptavidin-ALP (Mabtech) and BCIP/NBT (Thermo, UK). Plates were scanned using ImmunoSpots reader (AID, UK). The mean values were represented as spot-forming cells (SFCs) per 10^6^ cells. Levels were considered positive if at least two times above the background control.

### Tumour Models

The mouse B16F10 cell line expressing the human HLA-A2 gene (B16F10-HLA-A2) was provided by Professor Eric Tartour (Sorbonne Paris Cité, University). B16 is one of the most frequently used model for evaluation of the efficacy of anti-PD1 antibody and its combinations hence it has been chosen for the currently study in favor of less well characterized HNSCC models which has recently emerged (34). It was cultured in RPMI 1640 (Gibco, UK) supplemented with 10% heat inactivated-fetal bovine serum (FBS, Gibco UK), penicillin/streptomycin (100 U/ml) and 1 mg/ml G418 (EMD Millipore, US) at 37°C in a 5% CO_2_ humidified atmosphere. We generated two different tumor models expressing our target antigens; B16F10-HLA-A2-MAGED4B (BAM) and B16F10-HLA-A2-FJX1 (BAF). The expression of HLA-A2 in BAM and BAF cell lines was confirmed by flow cytometry using human HLA-A2–PE-conjugated antibody (clone BB7.2, BD Biosciences, US). MAGED4B and FJX1 expression levels were confirmed by western blotting using custom made anti-MAGED4B (Dundee Cell Products Ltd, UK) and anti-FJX1 (HPA059220, Sigma Aldrich, US) antibodies respectively.

### Efficacy of pDom-M/F *In Vivo*

AAD mice (transgenic for HLA-A2.1/H2-Dd allele) used in the efficacy study were bred in the animal facility of National University Malaysia. AAD mice (6-10 weeks) were inoculated with 1x10^6^ BAM cell line (n=27 mice) at the right flank at day 0. These mice were randomized to receive vaccination of 100 µg pDom-M/F DNA vaccines (50 µg of each vaccine) or 50 µg pDom DNA vaccine i.m into each quadriceps muscles of the hind legs on day 5 after palpable tumors were observed (~25cm^3^). A booster vaccination at the same dose was administered at day 12 (the vaccination schedule is depicted in Fig. 3A). Tumour sizes were evaluated every 3-4 days and volumes were calculated using the formula: volume = ½ (length X width^2^). Tumour growth inhibition (TGI) was calculated using the formula: TGI=(Vc-Vt)/Vc X 100%; where Vc and Vt are the average tumor volume of control and treatment group respectively at endpoint. On day 26, mice were sacrificed and tumors were harvested for analysis of T cell infiltration by IHC and flow cytometry as described below.

To evaluate the efficacy of combination treatment of pDom-M/F vaccine with anti-PD-1 antibody, AAD mice were inoculated with 1x10^5^ BAM (n=32 mice) or BAF cells (n=39 mice). Subsequently, mice were randomized into four treatment groups; pDom-M/F, anti-PD-1 antibody, pDom-M/F + anti-PD-1 antibody, or pDom + IgG isotype control. pDom-M/F vaccinations were given at day 5 and day 26 post-cell inoculation. One hundred microgram of anti-PD-1 monoclonal antibody (CD279, Bioxcell, US) or rat IgG2a isotype control (Bioxcell, US) was given intraperitoneally every 3 days from day 3 as indicated in **Figure 4A**. Tumour sizes were measured every 3-4 days and tumor volume were calculated using the formula described above.

### Flow Cytometry Analysis of Mouse Tumour Infiltrating Lymphocytes

To determine the expression of T cell markers after pDom-M/F treatment in mouse models, tumor infiltrating lymphocytes (TILs) were isolated from mouse tumor samples. Briefly, 500mm^3^ tumors were harvested and minced into smaller pieces (<3mm) and digested in 5 ml digestion buffer [RPMI 1640 containing collagenase type I (200 U/ml; Gibco, US) and DNase I (10 U/ml; Invitrogen, US)] and incubated at 37°C for 20 min with agitation. After incubation, tumor suspension was pressed through a 40 µm cell strainer, and rinsed with cold MACS buffer (0.5% BSA+2mM EDTA in PBS). Cells were spun at 500g for 5 min and finally resuspended in PBS with purified rat anti-mouse CD16/CD32 (mouse Fc blocker) (BD Pharmingen US), and proceed to stain with viability dye (FVS780), anti-CD4, CD8 and PD-1 antibodies (all from BD Biosciences, US) for 30 min at 4°C. To determine the presence of MDSC and T-regs, additional tubes were stained with viability dye (FVS780), anti-CD4, CD45.2, CD11b, and GR1. Cells were subjected to fixation and permeabilization with Mouse FoxP3 Buffer Set (BD Biosciences, US), and stained with anti-FOXP3 (eBioscience, US). Details of all antibodies used in the flow cytometry staining is provided in **Table S1**. PD1-positive CD4+ or CD8+ T cells were quantitated based on the relative expression of PD1, gating strategy is detailed in **Figure S3**.

### Immunohistochemistry

To assess the expression level of MAGED4B and FJX1 in HNSCC samples from the UK cohort, anti-MAGED4B polyclonal antibody (1:200, Novus-Bio) and anti-FJX1 polyclonal antibody (1:200, Novus-Bio) were used. Deparaffinization, rehydration, antigen retrieval, and IHC staining were performed using a Dako PT Link Autostainer using EnVision FLEX Target Retrieval Solution, High pH (Agilent Dako, UK) and DAKO Auto-stainer Link48™ in the Cellular Pathology Department of the University Hospital of Southampton NHS Trust. Images were captured using ZEISS Axio scanner.

For the Malaysian cohort, IHC was performed on patient FFPE samples using anti-MAGED4B polyclonal antibody (1:100, Sigma Aldrich, US) and anti-FJX1 polyclonal antibody (1:200, Sigma Aldrich, US). Antigen retrieval was performed in citrate buffer pH 6 and Tris-EDTA pH 9 for MAGED4B and FJX1 respectively using microwave heating method. IHC staining were performed using Dako Cytomation Envision+ Dual Link System- HRP (DAB+) kit (Dako,US) following protocol recommended by manufacturer. Direct comparisons of antibodies to each antigen used for the UK and the Malaysian cohorts were performed using 5 independent HNSCC samples and the same data were obtained. The Novus antibodies were chosen in Southampton because they generated less background with the automated system.

For mouse IHC staining, FFPE sections were stained with rabbit anti-mouse CD8α (1:400; clone D4W2Z; Cell Signaling Technologies, US). All sections from mice were processed after antibody staining using Dako Animal Research Kit (Dako, US) according to the recommendation by manufacturer (27). The field containing the highest density of CD8-positive cells within the tissue were identified. CD8-positive cells were counted by 3 individuals including a board-certified pathologist and graded as “less than 5 cells”, “5 to 10 cells” and “more than 10 cells” (35, 36).

### Statistics

Data are shown as the mean ± SD or median ± interquartile as described in the figure legends. Statistical differences in the expression of MAGED4B and FJX1 mRNA levels between tumor and normal samples in TCGA were determined using Kruskal Wallis test. ELISpot and FACS results analysis were performed using the Mann-Whitney test for non-parametric data. Tumour volume data was analyzed using the two-way repeated measures ANOVA by time and treatment using GraphPad PRISM as indicated in the figure legends.

## Results

### MAGED4B and FJX1 Are Expressed in HNSCC Tumour Samples

**Figure 1:**
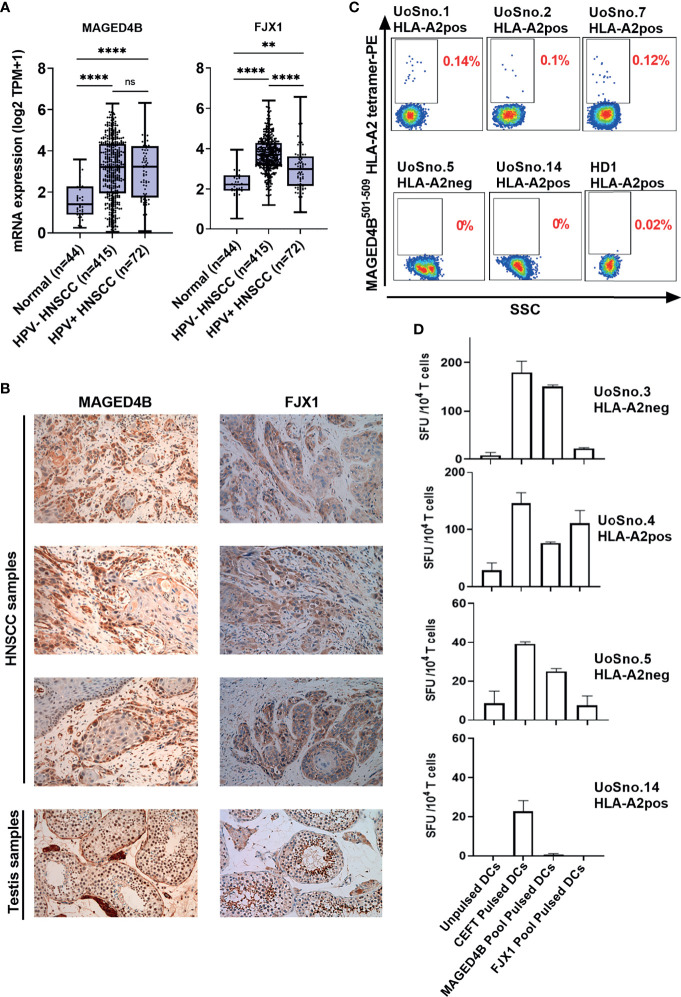
MAGED4B and FJX1 are immunogenic tumour-associated antigens in HPV^neg^ HNSCC patients. **(A)** Both HPV^neg^ and HPV^pos^ HNSCC samples from TCGA has significant elevation of MAGED4B and FJX1 compared to adjacent normal tissue at transcriptomic level. P values were calculated using Kruskal-Wallis test on normalized mRNA expression (****p-value < 0.0001; **p-value < 0.01; ns, not significant). **(B)** MAGED4B and FJX1 expression in HNSCC samples and testes (as positive control) were indicated by IHC staining with anti-hMAGED4B polyclonal antibody (Novus-Bio) and anti-FJX1 polyclonal antibody (Novus-Bio) respectively. Magnification x200. **(C)** FACS plots showed MAGED4B -specific T cells were detected in PBMC from HNSCC patients using MAGED4B^501-509^ HLA-A2 tetramer-PE. Four HLA-A2^pos^, one HLA-A2^neg^ HNSCC patients and one healthy donor are shown. **(D)** T cells from HNSCC patients after IVS were assessed with IFNγ ELISpot. Antigen-specific T cell responses were evaluated by stimulating with MAGED4B OPP and FJX1 OPP pulsed autologous DCs. CEFT OPP were used as positive control in IFNγ ELISpot. Two HLA-A2^pos^ and two HLA-A2^neg^ HNSCC patients are shown.

Using TCGA-HNSC project data, we demonstrated that both MAGED4B and FJX1 gene expression levels in the HPV negative (n=415) and HPV positive (n=72) tumour tissue samples were significantly higher than levels in adjacent normal tissues (n=44, **Figure 1A**). HPV status of TCGA samples was available from cBioportal for the PanCancer and was the result of complete analysis of the cohort for HPV transcripts (37). Importantly, either one or both antigens were also found expressed in other cancer types including lung, breast, oesophageal, and stomach cancer at the mRNA level (**Figure S4**). We subsequently examined the protein expression of MAGED4B and FJX1 in HNSCC tumour tissues from both Malaysian and UK cohorts by IHC. All tested samples from Malaysian cohort (n=28) expressed both antigens, except one patient who did not express FJX1 (**Table 1**). All samples from the UK cohort (n=16) expressed both MAGED4B and FJX1 (**Table 1**). In total 43/44 samples were positive for both antigens (representative images are depicted in **Figure 1B**; testes used as positive control). The evaluation of expression of MAGED4B and FJX1 in five major organs and healthy oral epithelia revealed very low levels in basal keratinocytes of stratified squamous epithelium with no expression above the background elsewhere (**Figure S5**).

### MAGED4B and FJX1 Specific T Cells Were Detected in HNSCC Patients at High Frequency

T cells specific for neo-antigens were detected in patients with HPV^neg^ HNSCC (38), indicating that the patients’ immune system is capable of recognising the tumour antigens in this disease. To determine whether HNSCC patients inherently harbour MAGED4B- and FJX1-specific T cells, we generated MAGED4B^501-509^/HLA-A2 tetramer using the HLA-A2 epitope we previously identified (39). MAGED4B-specific CD8+T cells were identified in 4/4 HLA-A2^pos^ patients from the Malaysian cohort and 8/11 HLA-A2^pos^ patients from the UK cohort (**Figure 1C** and **Table 2**) at a frequency observed at a similar level to neoepitopes in melanoma (range 0.06-0.12%) (40). PBMC samples of HLA-A2^neg^ patients were used to confirm the specificity (**Figure 1C**, **Figure S1B** and **Table 2**). Since no FJX1 epitope was available for a tetramer, the T cell responses were analysed by IFNγ ELISpot using OPP for the entire antigenic sequence. In parallel we also analysed the responses to MAGED4B OPP to extend the data beyond the HLA-A2 positive cohort. We initially performed *ex vivo* ELISpot using PBMCs from two patients (**Figure S1C**), which suggested specific T cell responses could be captured without *in vitro* re-stimulation (IVS) occasionally but not reliably. We therefore opted for IVS ELISpot; 3/7 and 5/7 patients responded to FJX1 OPP or MAGED4B OPP respectively with 3/7 demonstrating responses against both (**Figure 1D**; representative examples, **Table 2**). Collectively, we have confirmed both antigens are immunogenic in HPV^neg^ HNSCC patients.

**Table 2:** Information of target antigen expression, HLA type, and detection of antigen-specific T cells in UK and Malaysia patient cohorts.

Patient ID	Antigen expression (IHC)		HLA-A2	MAGED4B Tetramer+	ELISpot	
	MAGED4B	FJX1			MAGED4B	FJX1
UoSno.1	Pos	Pos	Pos	Neg	Pos	Pos
UoSno.2			Pos	Pos		
UoSno.3			Neg	Neg	Pos	Neg
UoSno.4	Pos	Pos	Pos	Pos	Pos	Pos
UoSno.5	Pos	Pos	Neg	Neg	Pos	Neg
UoSno.6	Pos	Pos	Pos	Pos		
UoSno.7	Pos	Pos	Pos	Pos		
UoSno.8	Pos	Pos	Pos	Pos		
UoSno.9	Pos	Pos	Neg			
UoSno.10	Pos	Pos	Pos	Pos		
UoSno.11	Pos	Pos	Pos	Pos		
UoSno.12	Pos	Pos	Neg	Neg		
UoSno.13			Pos	Pos	Pos	Pos
UoSno.14	Pos	Pos	Pos	Neg	Neg	Neg
UoSno.15			Pos	Neg		
UoSno.16			Neg	Neg	Neg	Neg
UoSno.17	Pos	Pos				
UoSno.18	Pos	Pos				
UoSno.19	Pos	Pos				
UoSno.20	Pos	Pos				
UoSno.21	Pos	Pos				
04-0017-17	Pos	Pos	Pos	Pos		
06-0008-19	Pos	Neg	Pos	Pos		
06-0011-19	Pos	Pos	Pos	Pos		
06-0012-19	Pos	Pos	Pos	Pos		
06-0005-19	Pos	Pos	Pos			
06-0037-17	Pos	Pos	Neg	Neg		
06-0019-17	Pos	Pos	Neg			
06-0024-19	Pos	Pos	Neg			
01-0010-17	Pos	Pos				
01-0002-18	Pos	Pos				
04-0018-17	Pos	Pos				
04-0019-17	Pos	Pos				
04-0020-17	Pos	Pos				
04-0001-18	Pos	Pos				
04-0003-18	Pos	Pos				
04-0005-18	Pos	Pos				
06-0014-11	Pos	Pos				
06-0047-17	Pos	Pos				
06-0048-17	Pos	Pos				
06-0053-17	Pos	Pos				
06-0008-18	Pos	Pos				
06-0016-18	Pos	Pos				
06-0021-18	Pos	Pos				
06-0025-18	Pos	Pos				
06-0033-18	Pos	Pos				
06-0038-18	Pos	Pos				
06-0003-19	Pos	Pos				
06-0014-19	Pos	Pos				

Blank space indicating assay was not processed. FFPE blocks for samples UoSno.2, 3, 13,15 and 16 were not available at the time of the study.

### DNA Vaccines Targeting MAGED4B and FJX1 Induce T Cell Responses in Mice

**Figure 2:**
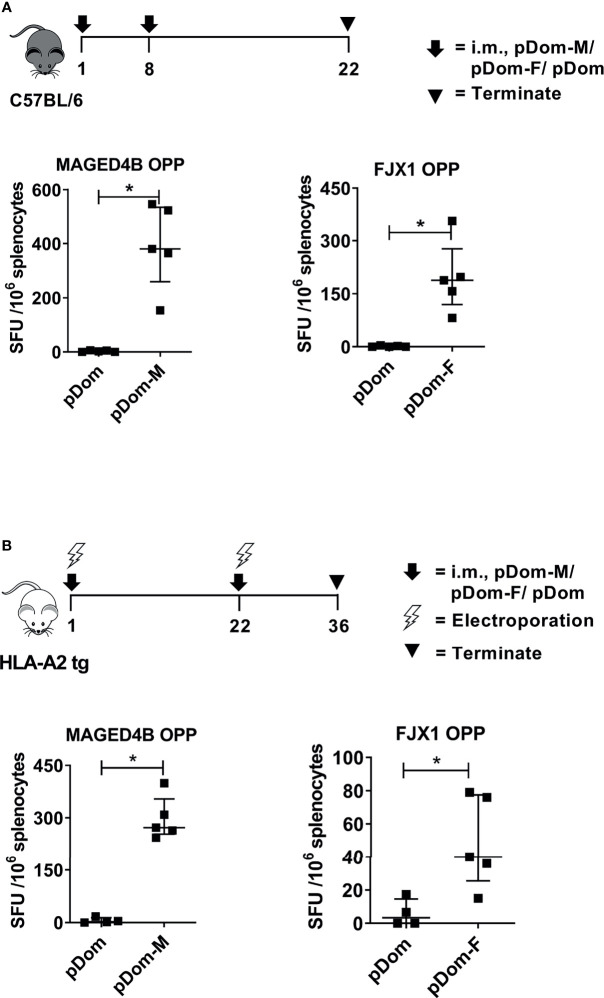
pDom-M and pDom-F vaccines are immunogenic in wildtype C57BL/6 and transgenic HLA-A2 mice. Augmentation of antigen-specific immune responses was observed in all mice that received pDom-M or pDom-F in comparison to mice which received pDom control. **(A)** C57BL/6 mice were vaccinated at day 1 and day 8 with pDom, pDom-M, or pDom-F. Splenocytes were harvested at day 22 to study antigen-specific immune responses by IFNγ ELISpot. **(B)** HLA-A2 tg mice were vaccinated at day 1 and day 22 with electroporation. Splenocytes were harvested at day 36 for IFNγ ELISpot. Splenocytes were seeded at 2.5 x10^5^ to ELISpot plates and stimulated overnight with 1 µM of MAGED4B OPP or FJX1 OPP. All data are expressed as mean ± SD. (*p-value < 0.05).

To induce broad CD4/CD8+ anti-tumor responses irrespective of HLA subtypes, the full-length MAGED4B or FJX1 sequences were linked to 3’ Dom sequence and inserted into pcDNA3 plasmid to give rise to pDom-MAGED4B (pDom-M, **Figure S2A**) and pDom-FJX1 (pDom-F, **Figure S2B**). *In vivo* immunogenicity was tested using the HLA-A2 transgenic (tg) and wildtype C57BL/6J mice using our previous protocols (31). Splenocytes from immunized mice were evaluated by IFNγ ELISpot using MAGED4B or FJX1 OPP to stimulate antigen-specific immune responses. In wildtype mice, pDom-M showed significantly higher antigen specific T cell responses to MAGED4B OPP than control pDom immunized mice (*p*=0.012, **Figure 2A**). Similarly, T cell response against FJX1 OPP was also significantly elevated in mice received pDom-F as compared to the pDom control group where no responses were detected (*p*=0.012). In HLA-A2 tg mice, vaccination with pDom-M and pDom-F resulted in a significant T cell response against MAGED4B (*p*=0.016, **Figure 2B**) or FJX1 (*p*=0.037, **Figure 2B**) OPP respectively as compared to the pDom control. Both vaccines induced robust T-cell responses with lower levels observed in HLA-A2 tg mice because of lower number of T-cells and lower MHC I expression (41). The responses to pDom vector control were negative in both strains as expected.

### Vaccination With pDom-M/F Vaccine Increased T Cell Infiltration and Delayed Tumour Growth

**Figure 3:**
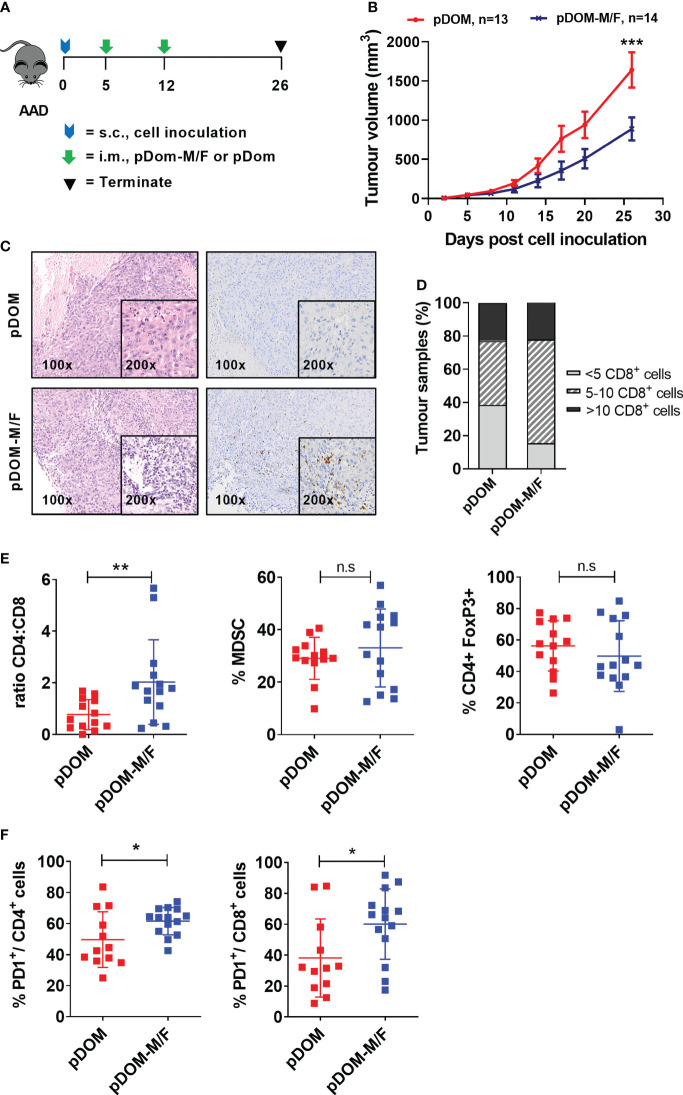
pDom-M/F vaccines enhance anti-tumour immunity by increasing infiltration of immune cells into the tumour. **(A)** Schematic of vaccination schedule, mice were inoculated with the BAM cell line and randomized to either received vaccination of pDom-M/F (pDom-M and pDom-F; 50 μg each vaccine per mouse) or pDom (50 μg per mouse) as vector control. Upon termination at endpoint, tumours from vaccinated animals were harvested for analysis of TILs. **(B)** Tumour growth inhibition was significant in mice that received pDom-M/F in comparison to mice which received pDom. Data is expressed as mean ± SEM. **(C)** Increased immune cells infiltration was observed in pDom-M/F group as opposed to pDom (left). IHC staining demonstrated CD8+ T cells were induced by pDom-M/F vaccination (right). Images shown were at 100X and 200X magnifications. **(D)** Analysis of IHC data demonstrated higher proportion of tumour from pDom-M/F group harbouring CD8+ T cells as compared to pDom vector control group. **(E)** pDom-M/F group significantly induced more CD4+ T cells. **(F)** Both PD1+CD4+ and PD1+CD8+ T cells were significantly upregulated in pDom-M/F vaccinated animals when compared to pDom group. Unless otherwise stated, all data are expressed as mean ± SD and pooled data from 2 independent experiments are shown for **(B–F)**. Symbols *, **, ***, ns denote *p* < 0.05, *p* < 0.01, *p* < 0.001 and not significant respectively.

We subsequently evaluated efficacy of these two vaccines using the BAM tumor model. This model expresses HLA-A2 (42) (**Figure S6A**). It expresses FJX1 endogenously and was engineered to express MAGED4B because the antigen is not expressed in mice (**Figure S6B**). The HLA-A2 mice were challenged with 10^6^ BAM tumor cells and once the tumors were palpable, mice were immunized with either combined pDom-M and pDom-F (referred as pDom-M/F vaccine) or pDom control as indicated (**Figure 3A**). Mice immunized with pDom-M/F showed a delay in their tumor growth as compared to mice received pDom (average tumor sizes of pDom and pDom-M/F at endpoint (day 26): 1641.22 mm^3^ and 887.24 mm^3^, TGI: 45.94%; **Figure 3B**).

Subsequently, we determined whether vaccination could increase infiltration of T cells into the tumor, as demonstrated in a DNA vaccine clinical trial targeting E6/E7 in HPV^pos^ HNSCC (18). Tumours from the pDom-treated group predominantly displayed an immune deserted phenotype, where immune cells were absent or scarce throughout the tissue (**Figure 3C**). This phenotype was expected as the parental tumor were previously reported to have low TILs (43). By contrast, tumors from mice vaccinated with pDom-M/F showed increased levels of T cells infiltrated into the tumor (**Figure 3C**). Enumeration of CD8+ T cells in the tumors further indicated that the infiltration of CD8+ T cells was higher in the pDom-M/F-vaccinated tumors as compared to pDom-vaccinated tumors (**Figure 3D**). As tumor-specific CD4 effector T cells can also contribute to anti-tumor protective mechanisms (44), we further determined whether they also increased. Encouragingly, pDom-M/F vaccine also induced CD4+ T cells in TILs as demonstrated by flow cytometry analysis but not MDSC (CD45.2+ CD11b+GR1+) and regulatory T cells (CD4+ FoxP3+) (**Figure 3E**). In addition, we detected a significant increase in PD-1 expressing CD4+ and CD8+ T cell population in the pDom-M/F group as compared to pDom (*p*=0.018 and *p*=0.029 respectively; **Figure 3F**). The increase in PD-1 expression level amongst CD4+ and CD8+ T cells indicated a combination with an anti-PD-1 antibody would likely to boost the T cell response.

Further, there was no significant difference in body weight between pDom and pDom-M/F treated animals and histopathology analysis of 5 major organs (kidney, lung, heart, spleen and liver) revealed no pDOM-M/F induced toxicity (data not shown).

### Combination Therapy of pDom-M/F and Anti-PD-1 Inhibited Tumour Growth

**Figure 4:**
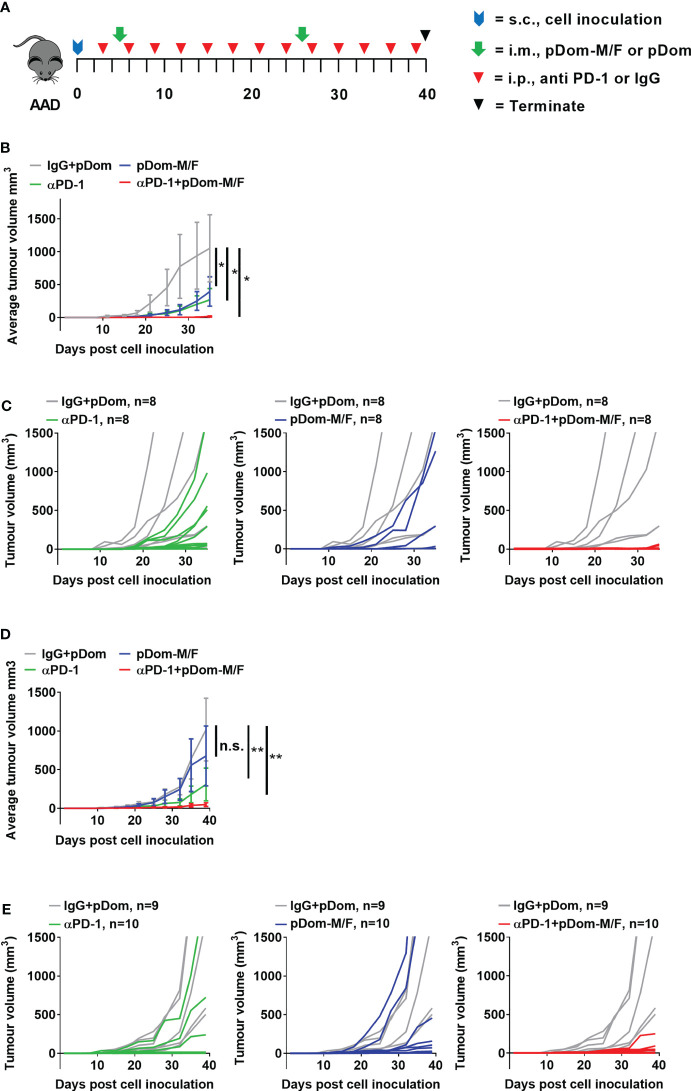
Combination therapy of pDom-M/F and anti-PD-1 inhibited tumour growth *in vivo*. **(A)** Schematic of vaccination schedule, mice were inoculated with the BAM or BAF cells and randomized to either received 2 doses of pDom (50 μg per mouse) as vector control combined with 13 doses of isotype control (100 μg per mouse, given every 3 days), 13 doses of anti-PD-1 (100 μg per mouse, given every 3 days), pDom-M/F (pDom-M and pDom-F; 50 μg each vaccine per mouse) and the combination of pDom-M/F with anti-PD-1. Upon termination at end-point, mouse spleens were harvested for ELISpot. **(B, D)** The graphs indicated mean tumour volumes of treatment group for BAM or BAF model respectively. Statistical analyses were conducted using with two-way ANOVA. (*p < 0.05; **p < 0.01; ns, not significant). Statistics represented the comparison on terminated date (day 35 for BAM, day 40 for BAF). **(C, E)** Graphs of individual animal tumour volumes for BAM and BAF models respectively comparing experimental groups including anti-PD-1 (green line), pDOM-M/F (blue line) and the combination of pDom-M/F with anti-PD-1 (red line) to IgG/pDOM control (grey line).

We next investigated if vaccination with pDom-M/F in combination with anti-PD1 antibody would lead to an improved tumor control. Anti-PD1 group served as a comparator while isotype control IgG plus pDom served as a negative control. When compared to IgG + pDom control group, growth inhibition was clearly demonstrated in pDom-M/F, anti-PD-1 and the combined treatment group (at day 35, TGI pDom-M/F: 49.16%, anti-PD-1: 60.11% and combination: 96.7%; **Figures 4B, C**). Significantly, 6 of 8 mice (75%) that received a combination of pDom-M/F and anti-PD-1 antibody had complete tumor growth inhibition.

We next used the BAF cells (engineered to overexpress human FJX1 only, **Figure S6C**), to evaluate the efficacy of pDom-M/F. The experiment was performed in BAF-bearing animals using the same protocol as for the BAM model. The growth inhibition in the combination group was also prominent (at day 40, TGI pDom-M/F: 37.78%, anti-PD-1: 71.59% and combination: 95.56%; **Figures 4D, E**). Fifty percent of animals (5 of 10) in the combination group had completely inhibited tumor growth throughout the experiment. We therefore were able to confirm that pDom-M/F DNA vaccines were able to significantly reduce the tumor burden and this was enhanced by combination with anti-PD1 antibody achieving a complete clearance of tumor in 50%-75% of mice in BAF and BAM model respectively.

## Discussion

Therapeutic vaccine targeting tumor antigens is a promising strategy to activate the immune system to eradicate cancer. This strategy confers advantage over other non-specific therapies, as it does not only induce tumor-specific immune responses, but also promotes establishment of immunological memory. Early trials in HNSCC had focused on targeting cancer driving mutations such as p53 and K-Ras, however no significant clinical outcomes were reported (45). Vaccines targeting MUC1 (NCT02544880) and CEA (NCT00924092; NCT00027534) which are in phase I/II clinical trials have yet to report results while the phase I study targeting Survivin-2B demonstrated low efficacy (46).

Several recent trials in solid tumors demonstrated significant immunogenicity (20–22, 25) with achieving partial or clinical remission. Remarkably, the antigens which have been targeted are not only mutated antigens for which the central tolerance is not expected but widely expressed antigens, tissue-specific antigens as well as cancer testis antigens (CTA) (25, 47, 48). The latter has long been thought of as “good” antigens due to their safety and immunogenicity; targeting NY-ESO1 and MAGE-A3 recently have demonstrated clinical benefits (25). However, most well-characterized CTAs are not expressed in a large proportion of head and neck cancer (49). Previously, the expression of MAGED4B and FJX1 CTAs has been described in HNSCC patients in Malaysia (26, 50, 51). Here we were able to confirm their expression at the RNA levels using 522 HNSCC samples deposited at TCGA. Through the collaborative effort of our Malaysian and UK teams, these findings have been further validated in two independent cohorts of patients in Malaysia (n=28) and the UK (n=16), confirming 43/44 patients are co-expressed FJX1 and MAGED4B in both primary and recurrent tumors. This is a remarkably high frequency not observed for other CTAs in HNSCC [reviewed in (52)]; our data also demonstrate consistency between several ethnic groups from the UK and Malaysia. Alcohol consumption and smoking are common etiological factors for HNSCC in both Malaysia and UK, while betel quid chewing is specific to the Malaysia cohort (53, 54). Since the causes of the disease are different, the discovery of antigens that are expressed in over 90% of patients is unexpected and confers a rare chance for the development of ‘off-the shelf’ cancer vaccine applicable worldwide. These antigens are relatively unexplored and therefore a very few defined epitopes are available (39, 50). For MAGED4B, we were able to detect the tetramer specific response in HLA-A2 patients (approx. 80%) with the levels that are similar to those reported for mutated antigens reassuring that the antigen is remarkably immunogenic in this patient cohort. We were not able to generate a tetramer using the only HLA-A2 epitope (11mer) from FJX1 described previously (50). We probed the immunogenicity of FJX1 using IVS ELISpot designed to expand memory T responses in PBMCs. Three out of eight patients generated responses to FJX1, confirming pre-existing memory T cell responses to FJX1. Notably, the same patients also demonstrated responses to MAGED4B.

T cell infiltration into tumor is an important criterion for a successful immunotherapy and is linked to better patient prognosis (13, 14). Several approaches including targeted therapy, radiotherapy and chemotherapy have also shown the ability to convert immunologically cold tumors into hot to increase response to checkpoint inhibitors (55–57), however these approaches are associated with treatment-associated side effects. We demonstrated in this study that the pDom-M/F vaccination were able to inhibit the growth of MAGED4B and FJX1-expressing tumors in HLA-A2 transgenic mice by approximately 50%. Importantly, pDom-M/F vaccination were able to convert cold B16 tumors into hot tumors with an increase of CD8+ T cells. This is encouraging as an effective vaccination in cancer therapy is frequently associated with high degree of cytotoxic T cells infiltration into the tumor. We were not able to determine if these are antigen specific CD8+ T cells as our HLA-A2 tetramer or the corresponding peptide was not presented in the HLA-A2 transgenic mice. The work is currently underway to define T-cell epitopes to allow further characterization of antigen-specific responses in murine models.

High expression of PD-1 on TILs has been shown to impair the anti-tumour immune responses in humans by engaging the PD-L1 and to inhibit TCR-mediated proliferation and cytokine production (58). The upregulation of PD-1 levels in vaccinated mice supports our strategy to combine the pDom-M/F vaccine with an anti-PD-1 antibody and this can potentially be applied in the clinic as anti-PD-1 antibodies have now been approved for the treatment of recurrent/metastatic HNSCC. Promisingly, we observed a complete tumour elimination or static tumour growth in the majority of mice that received both vaccine and anti-PD-1 antibody treatment. Our results are one of the few TAA-based DNA vaccines that show near complete tumour clearance in preclinical models when combined with the anti-PD-1 antibody. Overall, our study provides novel findings in which a DNA vaccine targeting TAAs frequently expressed in HNSCC is able to enhance the efficacy of anti-PD-1 therapy in preclinical settings. Clinical trials using selected epitopes including our own has focused on patients’ cohort expressing a particular HLA allele (most frequently A2 and A24) (47, 48, 59). Although we tested our vaccines in the HLA-A2 model, for clinical translation a full-length antigen vaccine is preferable to provide a population-wide coverage irrespective of HLA genotype (31).

This vaccine was designed with HPV^neg^ patients in mind, HPV^pos^ HNSCC patients may also benefit from this vaccine as the antigens are also expressed in these cancers. Both antigens are additionally co-expressed in non-small cell lung cancer (**Figure S4**), suggesting relevance of our approach for this common cancer. A phase I/II clinical trial testing our DNA vaccine delivered as doggybone DNA vaccine (33) in combination with anti-PD-1 antibody is due to begin recruitment of patients with recurrent HNSCC in the first half of 2022.

## Data Availability Statement

The original contributions presented in the study are included in the article/**Supplementary Material**. Further inquiries can be directed to the corresponding authors.

## Ethics Statement

The studies involving human participants were reviewed and approved by 1. The Institutional Review Board of the Faculty of Dentistry, University of Malaya; 2. Medical Research and Ethics Committee, Ministry of Health, Malaysia; 3. UK Medical Research and Ethics Committee; 4. Institutional approval at Southampton University Hospitals NHS Foundation Trust, Southampton, UK. The patients/participants provided their written informed consent to participate in this study. The animal study was reviewed and approved by 1. The Animal Ethics Committee of Universiti Kebangsaan Malaysia; 2. Animal Ethics Committee of University of Southampton.

## Author Contributions

Conception and design: KL, SCC, NS, CO, and GT. Development of methodology: CW, NSZ, SJC, CG, KL, SCC, and NS. Acquisition of data: CW, NSZ, SJC, JD, CG, NZ, BL, RS, EK, MTA, SI, SL, GT, KM, RZ and ZAR. Analysis and interpretation of data: CW, NSZ, SJC, CG, KL, SCC, and NS. Writing, review, and/or revision of the manuscript: All authors.

## Funding

This study is funded jointly by the Ungku Omar Fund and the Medical Research Council UK Newton fund (MR/PO13414/1), and internal funding from Cancer Research Malaysia.

## Conflict of Interest

KL and NS received funding from Touchlight Genetics Ltd for translation of the preclinical work into early phase clinical testing.

The remaining authors declare that the research was conducted in the absence of any commercial or financial relationships that could be construed as a potential conflict of interest.

## Publisher’s Note

All claims expressed in this article are solely those of the authors and do not necessarily represent those of their affiliated organizations, or those of the publisher, the editors and the reviewers. Any product that may be evaluated in this article, or claim that may be made by its manufacturer, is not guaranteed or endorsed by the publisher.

## References

[1] Bray F Ferlay J Soerjomataram I Siegel RL Torre LA Jemal A. Global Cancer Statistics 2018: GLOBOCAN Estimates of Incidence and Mortality Worldwide for 36 Cancers in 185 Countries. CA: Cancer J Clin (2018) 68(6):394–424. doi: 10.3322/caac.21492 30207593

[2] Wang C Dickie J Sutavani RV Pointer C Thomas GJ Savelyeva N. Targeting Head and Neck Cancer by Vaccination. Front Immunol (2018) 9:830. doi: 10.3389/fimmu.2018.00830 29740440 PMC5924779

[3] Warnakulasuriya S. Living With Oral Cancer: Epidemiology With Particular Reference to Prevalence and Life-Style Changes That Influence Survival. Oral Oncol (2010) 46(6):407–10. doi: 10.1016/j.oraloncology.2010.02.015 20403722

[4] Burtness B Harrington KJ Greil R Soulieres D Tahara M de Castro G Jr.. Pembrolizumab Alone or With Chemotherapy Versus Cetuximab With Chemotherapy for Recurrent or Metastatic Squamous Cell Carcinoma of the Head and Neck (KEYNOTE-048): A Randomised, Open-Label, Phase 3 Study. Lancet (2019) 394(10212):1915–28. doi: 10.1016/S0140-6736(19)32591-7 31679945

[5] Seiwert TY Burtness B Mehra R Weiss J Berger R Eder JP. Safety and Clinical Activity of Pembrolizumab for Treatment of Recurrent or Metastatic Squamous Cell Carcinoma of the Head and Neck (KEYNOTE-012): An Open-Label, Multicentre, Phase 1b Trial. Lancet Oncol (2016) 17(7):956–65. doi: 10.1016/S1470-2045(16)30066-3 27247226

[6] Ferris RL Blumenschein G Jr. Fayette J Guigay J Colevas AD Licitra L. Nivolumab for Recurrent Squamous-Cell Carcinoma of the Head and Neck. N Engl J Med (2016) 375(19):1856–67. doi: 10.1056/NEJMoa1602252 PMC5564292 27718784

[7] Patel SP Kurzrock R. PD-L1 Expression as a Predictive Biomarker in Cancer Immunotherapy. Mol Cancer Ther (2015) 14(4):847–56. doi: 10.1158/1535-7163.MCT-14-0983 25695955

[8] Davis AA Patel VG. The Role of PD-L1 Expression as a Predictive Biomarker: An Analysis of All US Food and Drug Administration (FDA) Approvals of Immune Checkpoint Inhibitors. J Immunother Cancer (2019) 7(1):278. doi: 10.1186/s40425-019-0768-9 31655605 PMC6815032

[9] Riaz N Havel JJ Makarov V Desrichard A Urba WJ Sims JS. Tumor and Microenvironment Evolution During Immunotherapy With Nivolumab. Cell (2017) 171(4):934–49 e16. doi: 10.1016/j.cell.2017.09.028 29033130 PMC5685550

[10] Ayers M Lunceford J Nebozhyn M Murphy E Loboda A Kaufman DR. IFN-Gamma-Related mRNA Profile Predicts Clinical Response to PD-1 Blockade. J Clin Invest (2017) 127(8):2930–40. doi: 10.1172/JCI91190 PMC5531419 28650338

[11] Havel JJ Chowell D Chan TA. The Evolving Landscape of Biomarkers for Checkpoint Inhibitor Immunotherapy. Nat Rev Cancer (2019) 19(3):133–50. doi: 10.1038/s41568-019-0116-x PMC6705396 30755690

[12] Jerby-Arnon L Shah P Cuoco MS Rodman C Su M-J Melms JC. A Cancer Cell Program Promotes T Cell Exclusion and Resistance to Checkpoint Blockade. Cell (2018) 175(4):984–97. e24. doi: 10.1016/j.cell.2018.09.006 30388455 PMC6410377

[13] Wood O Woo J Seumois G Savelyeva N McCann KJ Singh D. Gene Expression Analysis of TIL Rich HPV-Driven Head and Neck Tumors Reveals a Distinct B-Cell Signature When Compared to HPV Independent Tumors. Oncotarget (2016) 7(35):56781–97. doi: 10.18632/oncotarget.10788 PMC5302866 27462861

[14] Ganesan AP Clarke J Wood O Garrido-Martin EM Chee SJ Mellows T. Tissue-Resident Memory Features are Linked to the Magnitude of Cytotoxic T Cell Responses in Human Lung Cancer. Nat Immunol (2017) 18(8):940–50. doi: 10.1038/ni.3775 PMC6036910 28628092

[15] Ward MJ Thirdborough SM Mellows T Riley C Harris S Suchak K. Tumour-Infiltrating Lymphocytes Predict for Outcome in HPV-Positive Oropharyngeal Cancer. Br J Cancer (2014) 110(2):489–500. doi: 10.1038/bjc.2013.639 24169344 PMC3899750

[16] Chen DS Mellman I. Elements of Cancer Immunity and the Cancer–Immune Set Point. Nature (2017) 541(7637):321–30. doi: 10.1038/nature21349 28102259

[17] Ochoa de Olza M Navarro Rodrigo B Zimmermann S Coukos G. Turning Up the Heat on non-Immunoreactive Tumours: Opportunities for Clinical Development. Lancet Oncol (2020) 21(9):e419–e30. doi: 10.1016/s1470-2045(20)30234-5 32888471

[18] Aggarwal C Cohen RB Morrow MP Kraynyak KA Sylvester AJ Knoblock DM. Immunotherapy Targeting HPV16/18 Generates Potent Immune Responses in HPV-Associated Head and Neck Cancer. Clin Cancer Res (2019) 25(1):110–24. doi: 10.1158/1078-0432.Ccr-18-1763 PMC6320307 30242022

[19] Massarelli E William W Johnson F Kies M Ferrarotto R Guo M. Combining Immune Checkpoint Blockade and Tumor-Specific Vaccine for Patients With Incurable Human Papillomavirus 16-Related Cancer: A Phase 2 Clinical Trial. JAMA Oncol (2019) 5(1):67–73. doi: 10.1001/jamaoncol.2018.4051 30267032 PMC6439768

[20] Keskin DB Anandappa AJ Sun J Tirosh I Mathewson ND Li S. Neoantigen Vaccine Generates Intratumoral T Cell Responses in Phase Ib Glioblastoma Trial. Nature (2019) 565(7738):234–9. doi: 10.1038/s41586-018-0792-9 PMC6546179 30568305

[21] Sahin U Derhovanessian E Miller M Kloke BP Simon P Lower M. Personalized RNA Mutanome Vaccines Mobilize Poly-Specific Therapeutic Immunity Against Cancer. Nature (2017) 547(7662):222–6. doi: 10.1038/nature23003 28678784

[22] Ott PA Hu Z Keskin DB Shukla SA Sun J Bozym DJ. An Immunogenic Personal Neoantigen Vaccine for Patients With Melanoma. Nature (2017) 547(7662):217–21. doi: 10.1038/nature22991 PMC5577644 28678778

[23] Hilf N Kuttruff-Coqui S Frenzel K Bukur V Stevanovic S Gouttefangeas C. Actively Personalized Vaccination Trial for Newly Diagnosed Glioblastoma. Nature (2019) 565(7738):240–5. doi: 10.1038/s41586-018-0810-y 30568303

[24] Krauss J Krackhardt A Jager E Williams A Wold H Gerner L. Abstract CT217: An Open-Label, Phase I/IIa Study of VB10.NEO (DIRECT-01) in Combination With Checkpoint Blockade in Patients With Locally Advanced or Metastatic Solid Tumors Including Melanoma, NSCLC, Renal Cell Carcinoma, Urothelial Cancer or SSCHN. Cancer Res (2019) 79(13 Supplement):CT217–CT. doi: 10.1158/1538-7445.Am2019-ct217

[25] Sahin U Oehm P Derhovanessian E Jabulowsky RA Vormehr M Gold M. An RNA Vaccine Drives Immunity in Checkpoint-Inhibitor-Treated Melanoma. Nature (2020) 585(7823):107–12. doi: 10.1038/s41586-020-2537-9 32728218

[26] Chong CE Lim KP Gan CP Marsh CA Zain RB Abraham MT. Over-Expression of MAGED4B Increases Cell Migration and Growth in Oral Squamous Cell Carcinoma and is Associated With Poor Disease Outcome. Cancer Lett (2012) 321(1):18–26. doi: 10.1016/j.canlet.2012.03.025 22459352

[27] Chai SJ Fong SCY Gan CP Pua KC Lim PVH Lau SH. *In Vitro* Evaluation of Dual-Antigenic PV1 Peptide Vaccine in Head and Neck Cancer Patients. Hum Vaccin Immunother (2019) 15(1):167–78. doi: 10.1080/21645515.2018.1520584 PMC6363157 30193086

[28] Goot-Heah K Kwai-Lin T Froemming GR Abraham MT Nik Mohd Rosdy NM Zain RB. Human Papilloma Virus 18 Detection in Oral Squamous Cell Carcinoma and Potentially Malignant Lesions Using Saliva Samples. Asian Pac J Cancer Prevent: APJCP (2012) 13(12):6109–13. doi: 10.7314/apjcp.2012.13.12.6109 23464414

[29] Kreiter S Selmi A Diken M Sebastian M Osterloh P Schild H. Increased Antigen Presentation Efficiency by Coupling Antigens to MHC Class I Trafficking Signals. J Immunol (2008) 180(1):309–18. doi: 10.4049/jimmunol.180.1.309 18097032

[30] Rice J Elliott T Buchan S Stevenson FK. DNA Fusion Vaccine Designed to Induce Cytotoxic T Cell Responses Against Defined Peptide Motifs: Implications for Cancer Vaccines. J Immunol (2001) 167(3):1558–65. doi: 10.4049/jimmunol.167.3.1558 11466377

[31] Joseph-Pietras D Gao Y Zojer N Ait-Tahar K Banham AH Pulford K. DNA Vaccines to Target the Cancer Testis Antigen PASD1 in Human Multiple Myeloma. Leukemia (2010) 24(11):1951–9. doi: 10.1038/leu.2010.196 20861911

[32] Pascolo S Bervas N Ure JM Smith AG Lemonnier FA Perarnau B. HLA-A2.1-Restricted Education and Cytolytic Activity of CD8(+) T Lymphocytes From Beta2 Microglobulin (Beta2m) HLA-A2.1 Monochain Transgenic H-2Db Beta2m Double Knockout Mice. J Exp Med (1997) 185(12):2043–51. doi: 10.1084/jem.185.12.2043 PMC2196346 9182675

[33] Allen A Wang C Caproni LJ Sugiyarto G Harden E Douglas LR. Linear Doggybone DNA Vaccine Induces Similar Immunological Responses to Conventional Plasmid DNA Independently of Immune Recognition by TLR9 in a Pre-Clinical Model. Cancer Immunol Immunother: CII (2018) 67(4):627–38. doi: 10.1007/s00262-017-2111-y PMC5860099 29330557

[34] Wang Z Wu VH Allevato MM Gilardi M He Y Luis Callejas-Valera J. Syngeneic Animal Models of Tobacco-Associated Oral Cancer Reveal the Activity of *In Situ* Anti-CTLA-4. Nat Commun (2019) 10(1):5546. doi: 10.1038/s41467-019-13471-0 31804466 PMC6895221

[35] Koelzer VH Lugli A Dawson H Hadrich M Berger MD Borner M. CD8/CD45RO T-Cell Infiltration in Endoscopic Biopsies of Colorectal Cancer Predicts Nodal Metastasis and Survival. J Transl Med (2014) 12:81. doi: 10.1186/1479-5876-12-81 24679169 PMC4022053

[36] Miksch RC Schoenberg MB Weniger M Bosch F Ormanns S Mayer B. Prognostic Impact of Tumor-Infiltrating Lymphocytes and Neutrophils on Survival of Patients With Upfront Resection of Pancreatic Cancer. Cancers (Basel) (2019) 11(1). doi: 10.3390/cancers11010039 PMC6356339 30609853

[37] Bratman SV Bruce JP O’Sullivan B Pugh TJ Xu W Yip KW. Human Papillomavirus Genotype Association With Survival in Head and Neck Squamous Cell Carcinoma. JAMA Oncol (2016) 2(6):823–6. doi: 10.1001/jamaoncol.2015.6587 27010835

[38] Yang W Lee KW Srivastava RM Kuo F Krishna C Chowell D. Immunogenic Neoantigens Derived From Gene Fusions Stimulate T Cell Responses. Nat Med (2019) 25(5):767–75. doi: 10.1038/s41591-019-0434-2 PMC6558662 31011208

[39] Lim KP Chun NA Gan CP Teo SH Rahman ZA Abraham MT. Identification of Immunogenic MAGED4B Peptides for Vaccine Development in Oral Cancer Immunotherapy. Hum Vaccines Immunotherapeut (2014) 10(11):3214–23. doi: 10.4161/hv.29226 PMC4517457 25483651

[40] Gros A Parkhurst MR Tran E Pasetto A Robbins PF Ilyas S. Prospective Identification of Neoantigen-Specific Lymphocytes in the Peripheral Blood of Melanoma Patients. Nat Med (2016) 22(4):433–8. doi: 10.1038/nm.4051 PMC7446107 26901407

[41] Firat H Cochet M Rohrlich PS Garcia-Pons F Darche S Danos O. Comparative Analysis of the CD8(+) T Cell Repertoires of H-2 Class I Wild-Type/HLA-A2.1 and H-2 Class I Knockout/HLA-A2.1 Transgenic Mice. Int Immunol (2002) 14(8):925–34. doi: 10.1093/intimm/dxf056 12147629

[42] Pere H Montier Y Bayry J Quintin-Colonna F Merillon N Dransart E. A CCR4 Antagonist Combined With Vaccines Induces Antigen-Specific CD8+ T Cells and Tumor Immunity Against Self Antigens. Blood (2011) 118(18):4853–62. doi: 10.1182/blood-2011-01-329656 21908423

[43] Jong WY Bhattacharya S Yanamandra N Kilian D Shi H Yadavilli S. Tumor-Immune Profiling of Murine Syngeneic Tumor Models as a Framework to Guide Mechanistic Studies and Predict Therapy Response in Distinct Tumor Microenvironments. PloS One (2018) 13(11):e0206223. doi: 10.1371/journal.pone.0206223 30388137 PMC6214511

[44] Stevenson FK Ottensmeier CH Johnson P Zhu D Buchan SL McCann KJ. DNA Vaccines to Attack Cancer. Proc Natl Acad Sci USA (2004) 101 Suppl 2:14646–52. doi: 10.1073/pnas.0404896101 PMC521995 15292504

[45] Carbone DP Ciernik IF Kelley MJ Smith MC Nadaf S Kavanaugh D. Immunization With Mutant P53- and K-Ras-Derived Peptides in Cancer Patients: Immune Response and Clinical Outcome. J Clin Oncol (2005) 23(22):5099–107. doi: 10.1200/JCO.2005.03.158 15983396

[46] Miyazaki A Kobayashi J Torigoe T Hirohashi Y Yamamoto T Yamaguchi A. Phase I Clinical Trial of Survivin-Derived Peptide Vaccine Therapy for Patients With Advanced or Recurrent Oral Cancer. Cancer Sci (2011) 102(2):324–9. doi: 10.1111/j.1349-7006.2010.01789.x 21143701

[47] Chudley L McCann K Mander A Tjelle T Campos-Perez J Godeseth R. DNA Fusion-Gene Vaccination in Patients With Prostate Cancer Induces High-Frequency CD8(+) T-Cell Responses and Increases PSA Doubling Time. Cancer Immunol Immunother: CII (2012) 61(11):2161–70. doi: 10.1007/s00262-012-1270-0 PMC3493666 22729556

[48] McCann KJ Mander A Cazaly A Chudley L Stasakova J Thirdborough S. Targeting Carcinoembryonic Antigen With DNA Vaccination: On-Target Adverse Events Link With Immunologic and Clinical Outcomes. Clin Cancer Res (2016) 22(19):4827–36. doi: 10.1158/1078-0432.CCR-15-2507 PMC5330406 27091407

[49] Laban S Atanackovic D Luetkens T Knecht R Busch CJ Freytag M. Simultaneous Cytoplasmic and Nuclear Protein Expression of Melanoma Antigen-A Family and NY-ESO-1 Cancer-Testis Antigens Represents an Independent Marker for Poor Survival in Head and Neck Cancer. Int J Cancer (2014) 135(5):1142–52. doi: 10.1002/ijc.28752 24482145

[50] Chai SJ Yap YY Foo YC Yap LF Ponniah S Teo SH. Identification of Four-Jointed Box 1 (FJX1)-Specific Peptides for Immunotherapy of Nasopharyngeal Carcinoma. PloS One (2015) 10(11):e0130464. doi: 10.1371/journal.pone.0130464 26536470 PMC4633155

[51] Chai SJ Ahmad Zabidi MM Gan SP Rajadurai P Lim PVH Ng CC. An Oncogenic Role for Four-Jointed Box 1 (FJX1) in Nasopharyngeal Carcinoma. Dis Markers (2019) 2019:3857853. doi: 10.1155/2019/3857853 31236144 PMC6545767

[52] von Witzleben A Wang C Laban S Savelyeva N Ottensmeier CH. HNSCC: Tumour Antigens and Their Targeting by Immunotherapy. Cells (2020) 9(9). doi: 10.3390/cells9092103 PMC7564543 32942747

[53] Shaw R Beasley N. Aetiology and Risk Factors for Head and Neck Cancer: United Kingdom National Multidisciplinary Guidelines. J Laryngol Otol (2016) 130(S2):S9–S12. doi: 10.1017/s0022215116000360 27841107 PMC4873944

[54] Johnson DE Burtness B Leemans CR Lui VWY Bauman JE Grandis JR. Head and Neck Squamous Cell Carcinoma. Nat Rev Dis Primers (2020) 6(1):92. doi: 10.1038/s41572-020-00224-3 33243986 PMC7944998

[55] Apetoh L Ghiringhelli F Tesniere A Obeid M Ortiz C Criollo A. Toll-Like Receptor 4-Dependent Contribution of the Immune System to Anticancer Chemotherapy and Radiotherapy. Nat Med (2007) 13(9):1050–9. doi: 10.1038/nm1622 17704786

[56] Golden EB Chhabra A Chachoua A Adams S Donach M Fenton-Kerimian M. Local Radiotherapy and Granulocyte-Macrophage Colony-Stimulating Factor to Generate Abscopal Responses in Patients With Metastatic Solid Tumours: A Proof-of-Principle Trial. Lancet Oncol (2015) 16(7):795–803. doi: 10.1016/S1470-2045(15)00054-6 26095785

[57] Brody JD Ai WZ Czerwinski DK Torchia JA Levy M Advani RH. *In Situ* Vaccination With a TLR9 Agonist Induces Systemic Lymphoma Regression: A Phase I/II Study. J Clin Oncol (2010) 28(28):4324. doi: 10.1200/JCO.2010.28.9793 20697067 PMC2954133

[58] Freeman GJ Long AJ Iwai Y Bourque K Chernova T Nishimura H. Engagement of the PD-1 Immunoinhibitory Receptor by a Novel B7 Family Member Leads to Negative Regulation of Lymphocyte Activation. J Exp Med (2000) 192(7):1027–34. doi: 10.1084/jem.192.7.1027 PMC2193311 11015443

[59] Yoshitake Y Fukuma D Yuno A Hirayama M Nakayama H Tanaka T. Phase II Clinical Trial of Multiple Peptide Vaccination for Advanced Head and Neck Cancer Patients Revealed Induction of Immune Responses and Improved OS. Clin Cancer Res (2015) 21(2):312–21. doi: 10.1158/1078-0432.CCR-14-0202 25391695

