# Supplementary material for: DNA Vaccines Targeting Novel Cancer-Associated Antigens Frequently Expressed in Head and Neck Cancer Enhance the Efficacy of Checkpoint Inhibitor
Source: Front Immunol. 2021 Oct 18;12:763086. doi: 10.3389/fimmu.2021.763086 (PMC8559892; doi:10.3389/fimmu.2021.763086)
Supplement: Supplementary file 1 [file DataSheet_1.pdf]

Supplementary Figure S1

(A) MAGED4B$^{501\text{-}509}$/HLA-A2 tertramer-PE staining

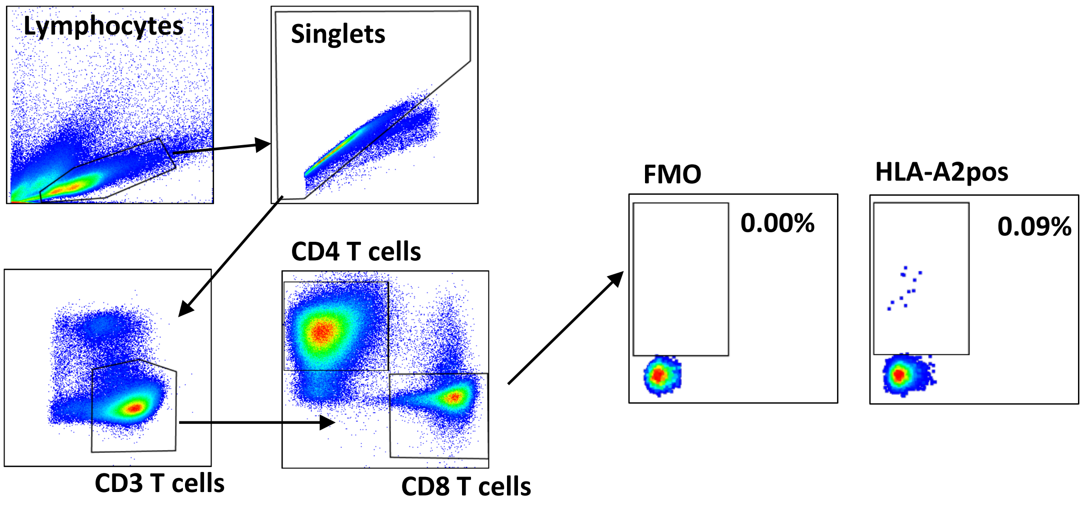

(B) *ex vivo* IFNγ ELIspots

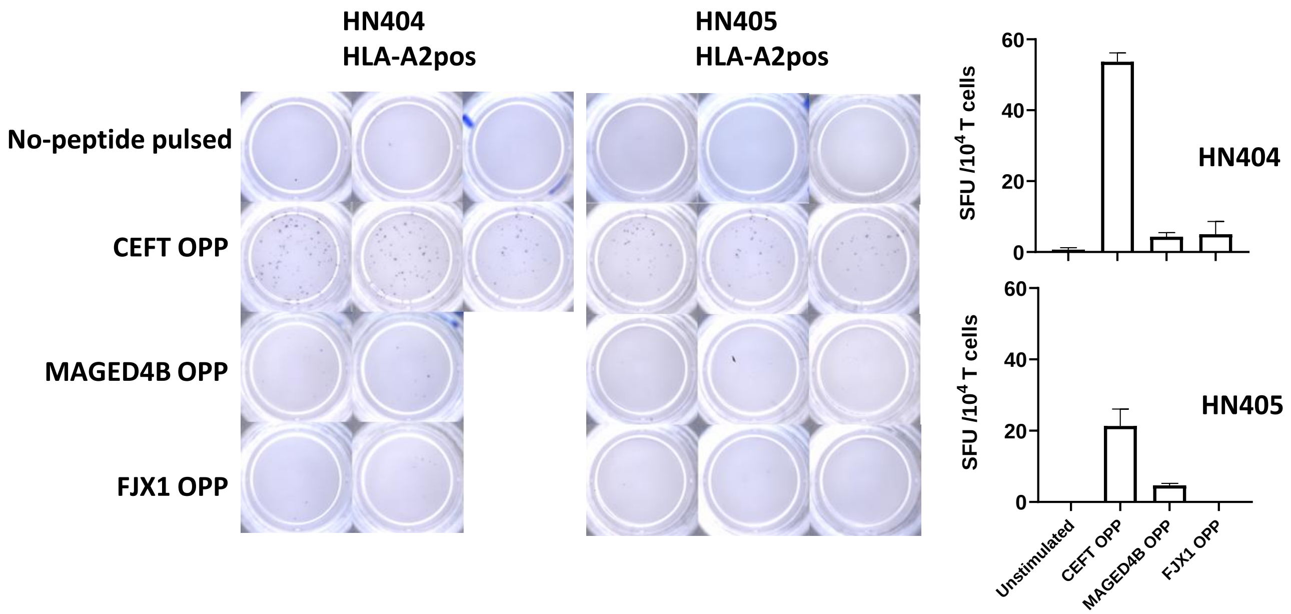

Supplementary Figure S1. The immunogenicity of MAGED4B and FJX1 were assessed by tetramer staining and IFN-γ ELISpots. (A) FACS **g**ating strategy of MAGED4B$^{501\text{-}509}$ /HLA-A2 tetramer-PE staining. (B) *Ex vivo* IFN-γ ELISpots, T cells isolated from PBMCs were incubated with irradiated T cells depleted PBMCs (idPBMCs) pulsed with 1 μg/ml (CEFT OPP, positive control; MAGED4B OPP; FJX1 OPP) at 1:1 ratio. Negative control was T cells incubated idPBMCs without stimulus. Spots more than 2X of negative control are considered as positive results.

Supplementary Figure S2

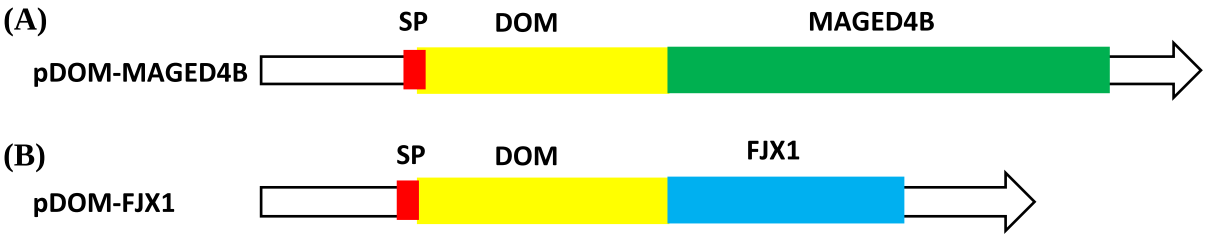

Supplementary Figure S2: Map of plasmid constructs used in this study. (A) pDom-M (B) pDom-F. SP represents leader signal peptide from mouse IgH signal peptide (MGWSCIIFFLVATATGVHS). DOM represents Dom sequence from fragment C of tetanus toxin. MAGED4B and FJX1 are the antigens of interest in this project.

Supplementary Figure S3

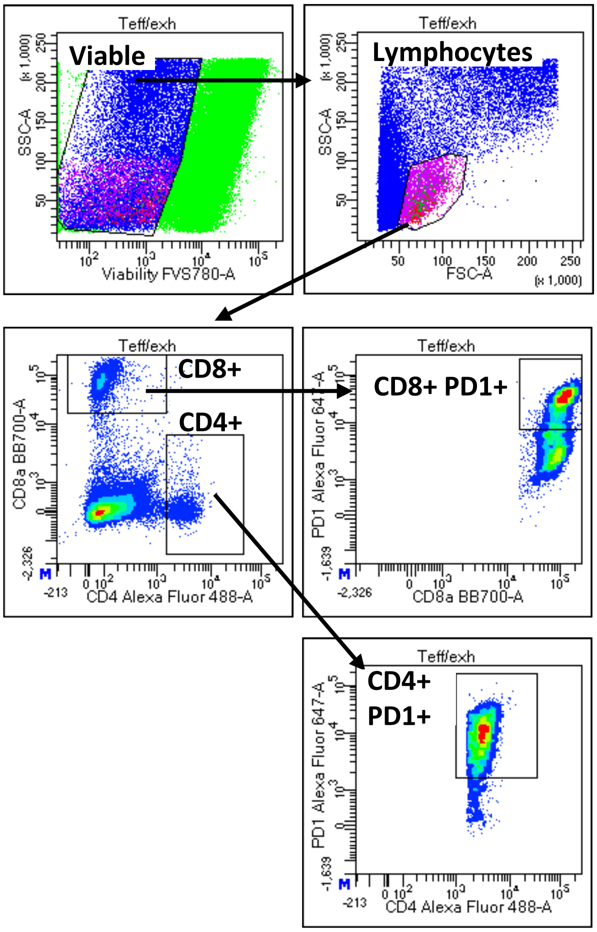

Supplementary Figure S3: Gating strategy for PD1-positive CD4+ and CD8+ T cells.

Supplementary Figure S4

(A)

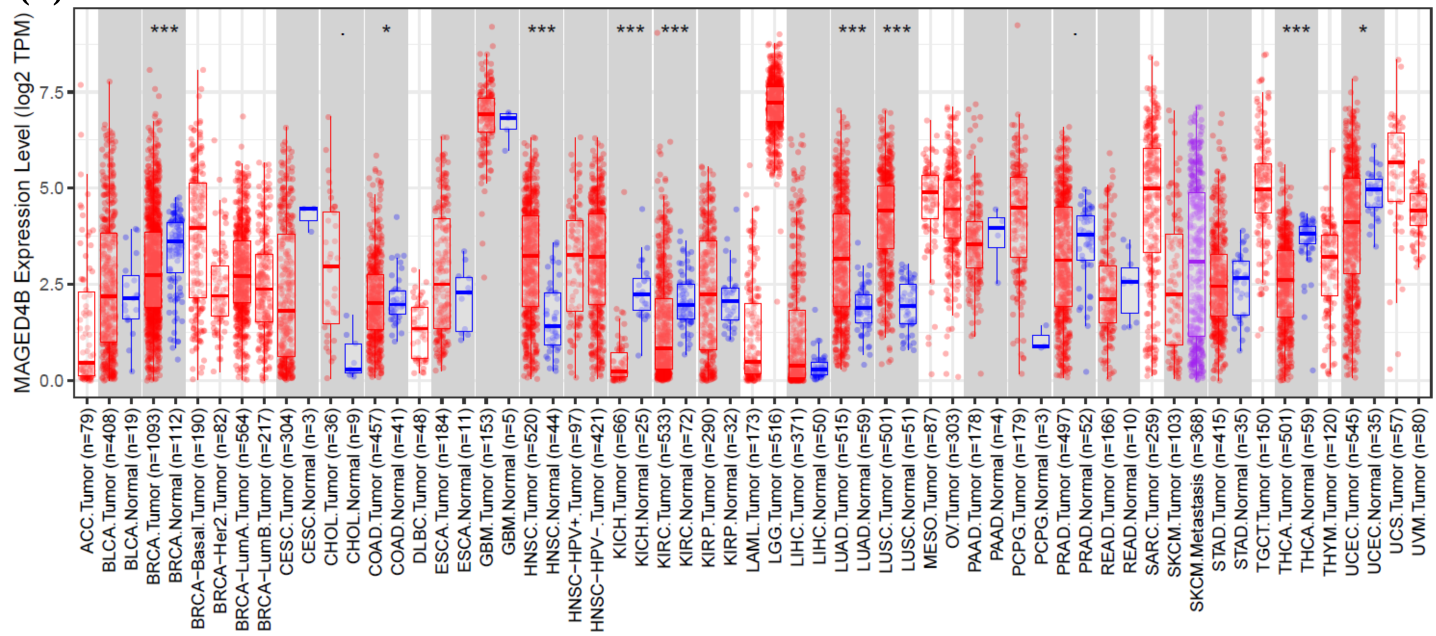

(B)

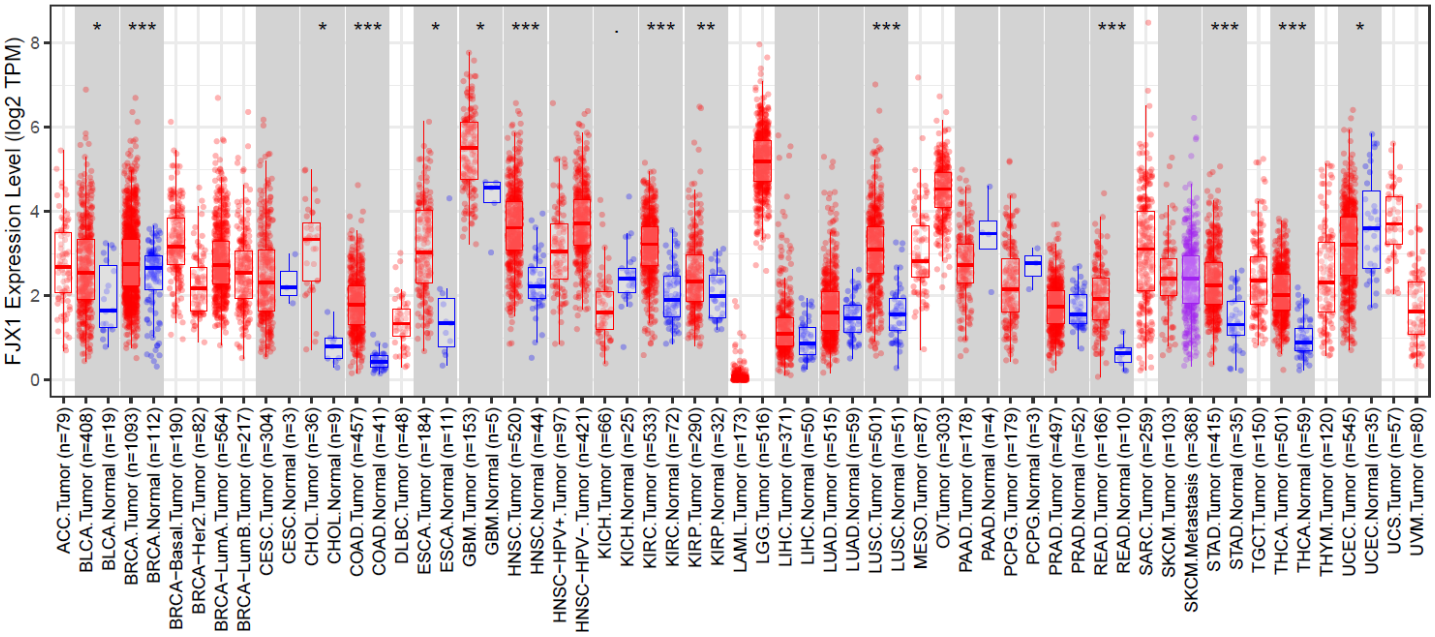

Supplementary Figure S4. MAGED4B and FJX1 were found overexpressing in tumour tissues from different types of cancers. The boxplot graphs were generated by Timer2.0 (http://timer.comp-genomics.org/) to study gene expression between tumour and adjacent normal tissues for MAGED4B (**A**), and FJX1 (**B**) across all 33 types of TCGA studies tumours. The statistical significance computed by differential analysis (edgeR) on RNA-Seq raw counts is annotated by the number of stars (*: p-value < 0.05; **: p-value <0.01; ***: p-value <0.001). Gray columns indicated when normal data are available to make the comparison.

Supplementary Figure S5

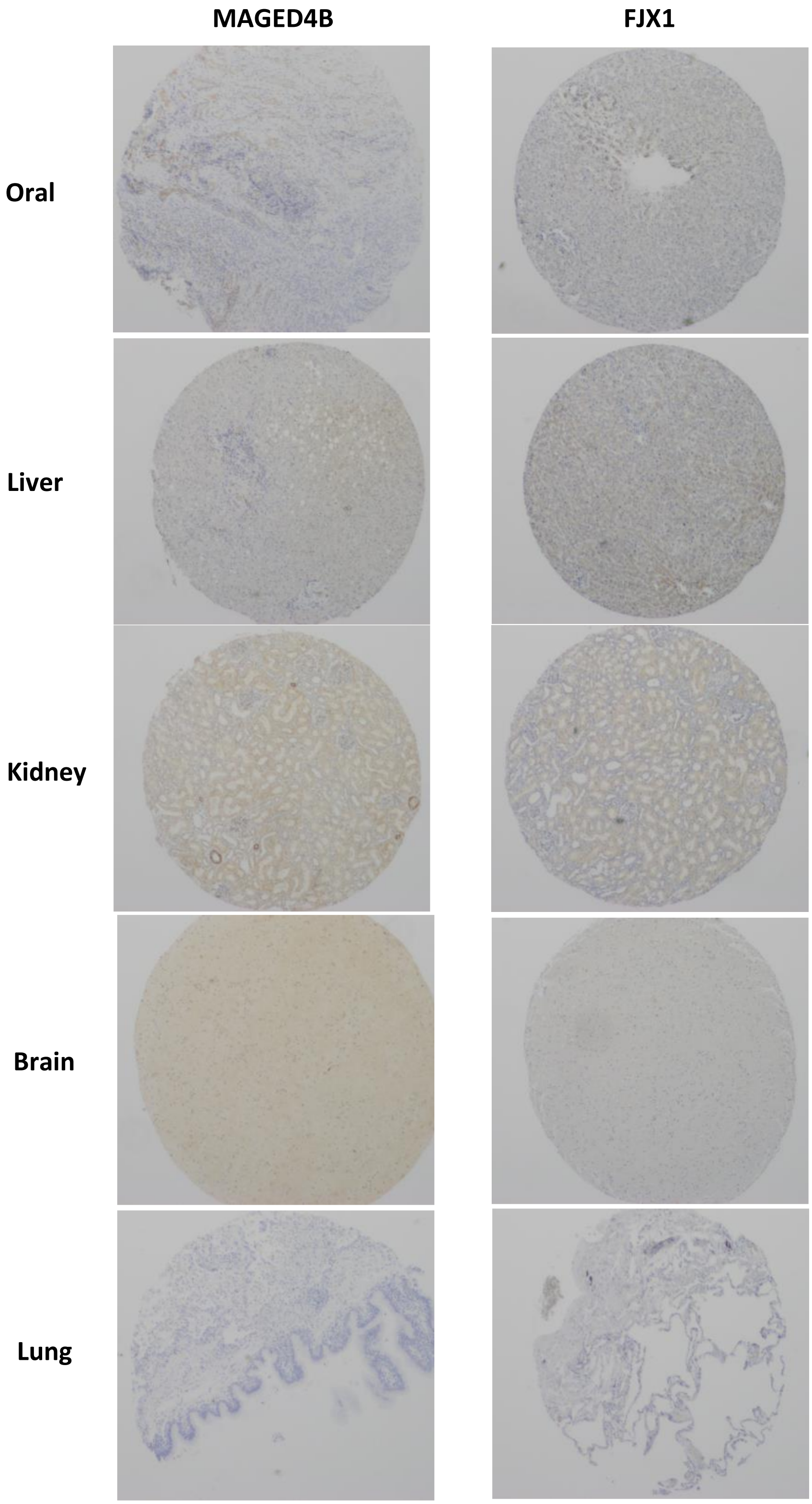

Supplementary Figure S5. MAGED4B and FJX1 were not expressed in five major organs. (A) TMAs were stained with MAGED4B (Novus-Bio:NBP1-89594) at 1:200 dilution. Staining was performed on a DAKO autostainer. Testis exhibits very strong staining for MAGED4B and was used as a positive control; secondary antibody only was used as a negative control. (B) TMAs were stained with FJX1 (Novus-Bio:NBP1-59470) at 1:100 dilution. Staining was performed on a DAKO autostainer. Testis exhibits very strong staining for FJX1 and was used as a positive control; secondary antibody only was used as a negative control.

Supplementary Figure S6

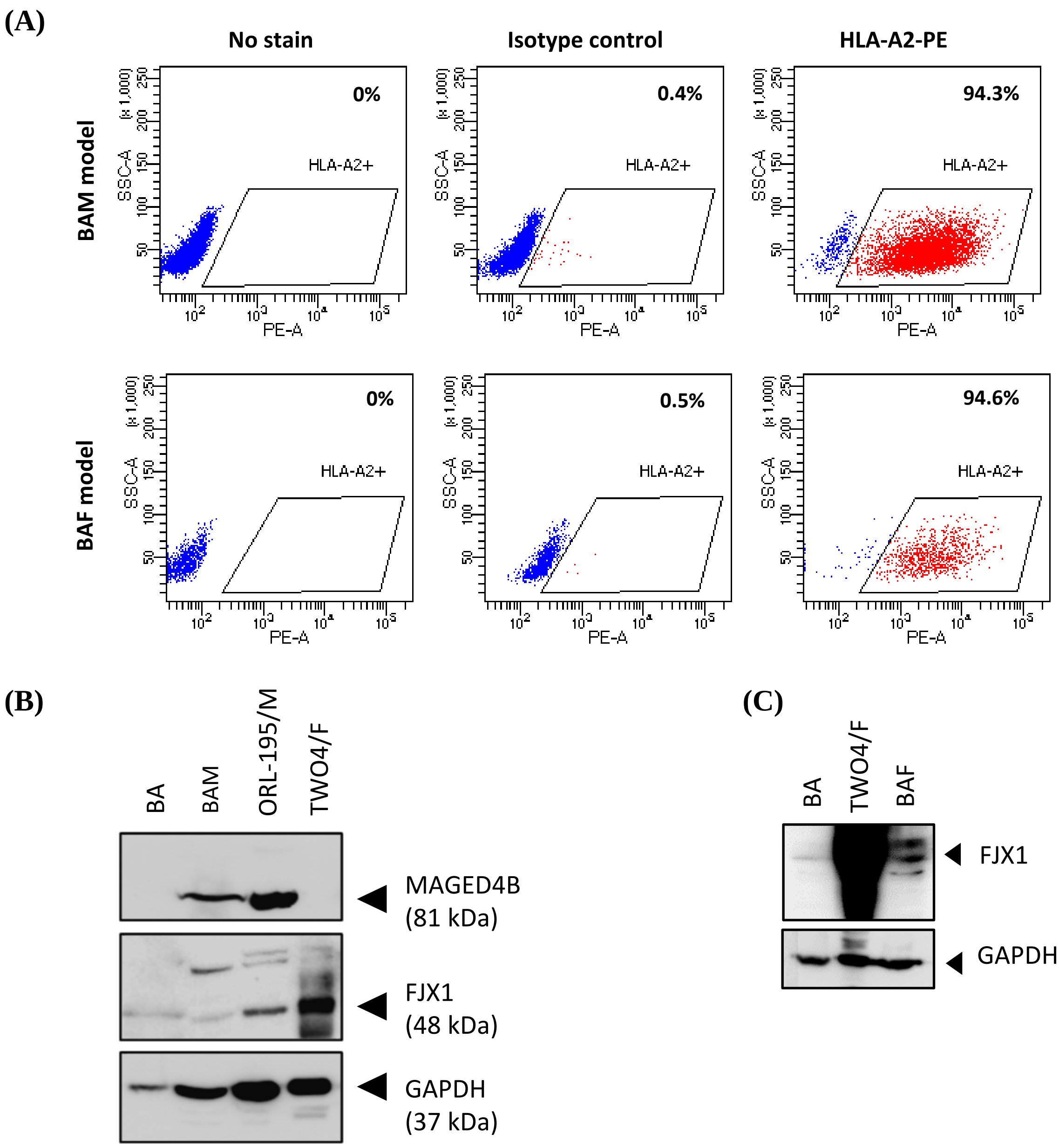

Supplementary Figure S6. Expression of HLA-A2 and the two target antigens, MAGED4B and FJX1 in BAM and BAF cell lines respectively. (A) Status of HLA-A2 in BAM and BAF cells were examined by flow cytometry. Cell lines were grown in culture, harvested, stained with HLA-A2-PE (clone BB7.2). Numbers in box represent the percentage of cell lines expressing HLA-A2 gated over single cells population. (B) Detection of human MAGED4B and endogenous murine FJX1 expression in BAM cell lines by western blotting. B16-F10 expressing HLA-A2 (BA) and ORL-195 overexpressing MAGED4B (ORL-195/M) were used as negative and positive controls for MAGED4B protein expression respectively. TW04 overexpressing FJX1 (TW04/F) was used as positive control for FJX1 expression. (C) Detection of FJX1 expression in BAF cell lines by western blotting.
