# Supplementary material for: DNA Vaccines Targeting Novel Cancer-Associated Antigens Frequently Expressed in Head and Neck Cancer Enhance the Efficacy of Checkpoint Inhibitor
Source: Front Immunol. 2021 Oct 18;12:763086. doi: 10.3389/fimmu.2021.763086 (PMC8559892; doi:10.3389/fimmu.2021.763086)
Supplement: Supplementary file 2 [file Table_1.docx]

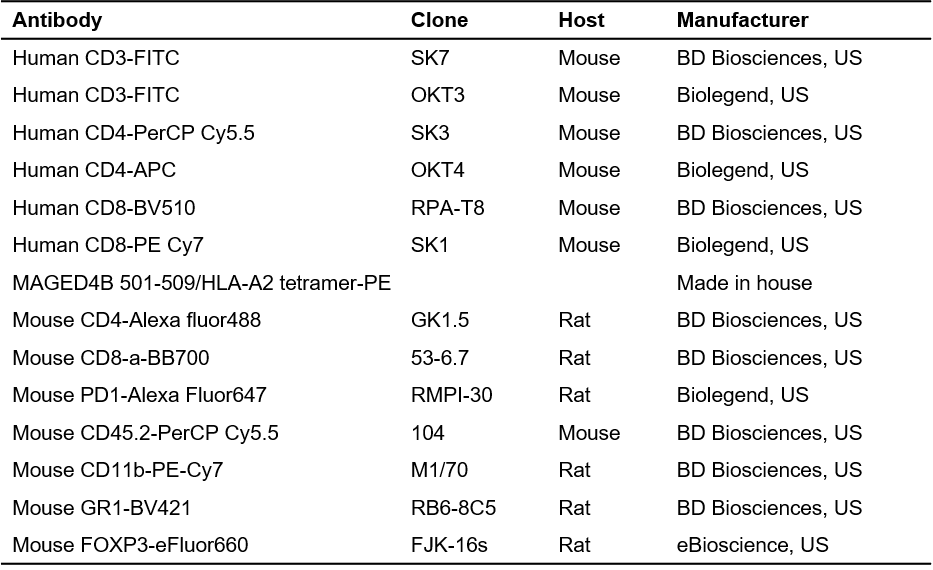


**Supplementary Table S1. List of antibodies used in flow cytometry analysis of HNSCC patients’ PBMC and mouse cells.**
